# Prognostic impact of body composition in hepatocellular carcinoma patients undergoing interventional and systemic therapy

**DOI:** 10.3389/fnut.2025.1586202

**Published:** 2025-04-16

**Authors:** Yun Feng, Bingran Yu, Anxiao Liu, Chongpeng Cai, Changming Zhou, Tong Tong, Lu Wang, Qi Pan

**Affiliations:** ^1^Department of Hepatic Surgery, Fudan University Shanghai Cancer Center, Shanghai, China; ^2^Department of Oncology, Shanghai Medical College, Fudan University, Shanghai, China; ^3^Department of Pediatric Surgery, Shanghai Key Laboratory of Birth Defects, Children's Hospital of Fudan University, Shanghai, China; ^4^Department of General Surgery, Shanghai Xuhui District Central Hospital, Shanghai, China; ^5^Department of Radiology, Fudan University Shanghai Cancer Center, Shanghai, China; ^6^Department of Cancer Prevention, Fudan University Shanghai Cancer Center, Shanghai, China

**Keywords:** sarcopenia, myosteatosis, hepatocellular carcinoma, hepatic arterial infusion chemotherapy, targeted therapy, immunotherapy

## Abstract

**Background:**

Primary liver cancer, predominantly hepatocellular carcinoma (HCC), is a leading cause of cancer-related deaths globally. Despite advances in targeted therapy and immunotherapy, survival rates for advanced HCC remain low. Combining hepatic artery infusion chemotherapy (HAIC) with systemic therapies shows potential, but identifying patients who benefit most is challenging. Body composition, including sarcopenia and myosteatosis, has been linked to cancer prognosis, but its role in HCC patients receiving HAIC with targeted and immunotherapies is unclear.

**Methods:**

This retrospective study analyzed 158 HCC patients treated with HAIC, tyrosine kinase inhibitors, and anti-PD-1 immunotherapy from January 2021 to October 2024. Body composition was assessed via CT scans at the L3 level, with sarcopenia defined by skeletal muscle index (SMI) and myosteatosis by skeletal muscle density (SMD). Progression-free survival (PFS) and overall survival (OS) were evaluated, and Cox regression analyses identified prognostic factors.

**Results:**

Sarcopenia cutoffs were 47.1 cm^2^/m^2^ (males) and 38.2 cm^2^/m^2^ (females); myosteatosis cutoffs were 40.8 HU (males) and 38.9 HU (females). Sarcopenic patients had lower BMI (*p* < 0.001) and higher ALBI scores (*p* = 0.006). Tumor response rates (ORR 53.4%, DCR 77.9%) were similar between sarcopenic and non-sarcopenic groups (*p* = 0.531 and *p* = 0.699). Myosteatosis showed no significant differences in ORR (54.0%) or DCR (77.0%) (*p* = 0.693 and *p* = 0.872). Median PFS did not differ between sarcopenic (9.53 months) and non-sarcopenic (13.87 months) patients (*p* = 0.536). However, sarcopenic patients had significantly shorter OS (20.80 vs. 35.97 months, *p* = 0.005). Myosteatosis also correlated with shorter OS (20.80 vs. 35.97 months, *p* = 0.021). Multivariate analysis identified sarcopenia as an independent risk factor for OS (HR: 0.527, *p* = 0.017), alongside AFP levels and tumor number.

**Conclusion:**

Sarcopenia and myosteatosis predict poor prognosis in HCC patients receiving HAIC with targeted therapy and immunotherapy. Sarcopenia is an independent risk factor for OS, highlighting the importance of body composition in prognosis. No significant associations were found between body composition and tumor response or PFS.

## Introduction

Primary liver cancer ranks as the sixth most prevalent cancer globally and the third leading cause of cancer-related deaths, with hepatocellular carcinoma (HCC) comprising about 85% of all cases (1). Although targeted therapy and immunotherapy have markedly enhanced survival outcomes for patients with advanced HCC, the overall survival (OS) rate continues to remain below 30% (2). As a result, there has been growing interest in investigating the integration of locoregional therapies, such as hepatic artery infusion chemotherapy (HAIC), with systemic approaches like targeted therapy and immunotherapy (3, 4). Despite the high rates of disease control and objective responses achieved by these combination strategies, identifying the specific populations that are most likely to benefit remains a significant challenge.

A range of prognostic biomarkers and staging systems has been established to predict treatment outcomes in patients with HCC. These include hematological markers such as alpha-fetoprotein (AFP) (5), the albumin-bilirubin (ALBI) ratio (6), the model for end-stage liver disease (MELD) score (7), the Barcelona Clinic Liver Cancer (BCLC) staging system (8), and the Child-Pugh score (9). However, most of these parameters focus on a single dimension of the disease, such as tumor characteristics or liver function, while failing to comprehensively assess the patient’s overall nutritional and metabolic status.

Recent research increasingly highlights the critical role of nutritional status in influencing tumor prognosis. Sarcopenia, characterized by reduced muscle quantity, and myosteatosis, indicative of compromised muscle quality, have been identified as key factors associated with adverse clinical outcomes in HCC patients receiving various therapeutic modalities (10). Despite these findings, there is a notable lack of studies specifically investigating the impact of body composition on the prognosis of HCC patients undergoing HAIC in combination with targeted therapy and immunotherapy.

This study retrospectively analyzed the body composition of HCC patients receiving HAIC in combination with targeted therapy and immunotherapy, with the aim of evaluating the prognostic significance of both sarcopenia and myosteatosis in this population.

## Materials and methods

### Patients

This retrospective study was conducted in accordance with the Declaration of Helsinki and approved by the Institutional Review Board of Fudan University Shanghai Cancer Center (FUSCC). Between January 2021 and October 2024, patients diagnosed with HCC who received HAIC combined with targeted therapy (tyrosine kinase inhibitors, TKIs) and immunotherapy (anti-programmed cell death 1, anti-PD-1) agents from Department of Hepatic Surgery were reviewed for eligibility. Patients were included based on the following criteria: age ≥ 18 years; Karnofsky performance score (KPS) ≥ 80; Child-Pugh score A/B liver function; at least one measurable intrahepatic lesion according to the modified Response Evaluation Criteria in Solid Tumors (mRECIST) (11); and adequate organ function [absolute neutrophil count ≥ 1.2 × 10^9^/L, platelet count ≥ 60 × 10^9^/L, total bilirubin (T-BIL) < 30 μmol/L, albumin (ALB) ≥ 30 g/L, aspartate transaminase (AST) and alanine transaminase (ALT) ≤ 5 × upper limit of the normal, creatinine clearance rate of ≤ 1.5 × upper limit of the normal, and left ventricular ejection ≥ 45%]. Patients were excluded if they had received other treatments for HCC during combination therapy. Patients were also excluded if they were diagnosed with other malignant tumors or if their medical information and follow-up data were incomplete.

### Treatment procedures

The Seldinger technique was used to puncture the femoral artery and a catheter was inserted into the feeding hepatic artery under digital subtraction angiography guidance. The chemotherapeutic regimen of HAIC (oxaliplatin 85/m^2^ from hours 0 to 2 on day 1; leucovorin 400 mg/m^2^ from hours 2 to 3 on day 1; 5-fluorouracil 400 mg/m^2^ bolus at hour 3; and 2,400 mg/m^2^ over 46 h on days 1 and 2) was infused via the catheter in the ward (4). HAIC was repeated every 3 weeks. TKIs [lenvatinib: a dose of 12 mg/day (for body weight ≥ 60 kg) or 8 mg/day (for body weight < 60 kg); apatinib, a dose of 250 mg/day; donafenib, a dose of 200 mg twice daily] were initiated on the first day following the initial interventional treatment, and discontinued from 1 day before each session of the interventional therapy to the time of withdrawal of the catheter. Anti-PD-1 antibodies (tislelizumab, 200 mg/3 weeks; sintilimab, 200 mg/3 weeks; toripalimab, 240 mg/3 weeks) were administered intravenously every 3 weeks. Dose reduction and treatment interruption depended on disease progression, unacceptable toxicity, patients’ withdrawal of consent or changes in treatment plan, and technical difficulties in repeating interventional therapy. Enhanced computed tomography (CT) or magnetic resonance imaging (MRI) was performed to evaluate therapeutic efficacy every 6 weeks after treatment initiation.

### Body composition assessment

**Figure 1:**
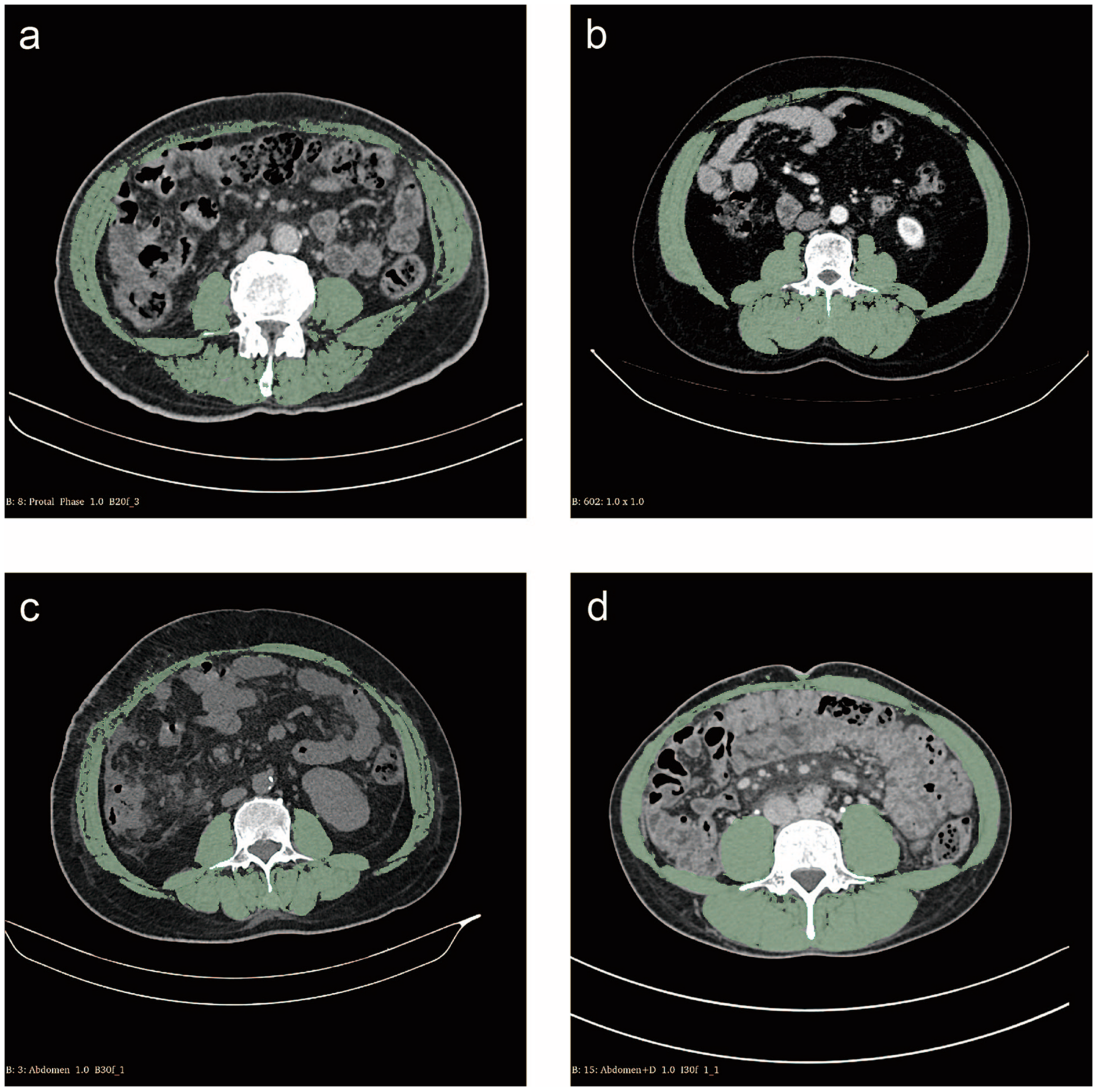
Cross-sectional computed tomography measurement of skeletal muscle areas (green) at the L3 vertebral layer. **(a)** Patient with sarcopenia of skeletal muscle index (SMI) 38.74 cm^2^/m^2^; **(b)** Patient without sarcopenia of SMI 61.99 cm^2^/m^2^; **(c)** Patient with myosteatosis of skeletal muscle density (SMD) 35.53 HU; **(d)** Patient without myosteatosis of SMD 56.62 HU.

Slice-O-Matic software (version 5.0; Tomovision, Montreal, Canada) was used by two researchers independently in a blinded fashion to identify and quantify skeletal muscle, subcutaneous adipose tissue, visceral adipose tissue and their total cross-sectional area at the L3 level in Hounsfield units (HU). The threshold range was defined as follows: skeletal muscle was defined as −29 HU to 150 HU, subcutaneous adipose tissue was defined as −190 HU to −30 HU, and visceral adipose tissue was defined as −150 HU to −50 HU (Figure 1) (12).

Body mass index (BMI) was calculated as weight (kg) divided by height (m^2^). According to the World Health Organization classification, a BMI of less than 18.5 is categorized as underweight, a BMI between 18.5 and 24.9 is considered normal weight, and a BMI of 25 or higher is classified as overweight (13). As an internationally recognized standard for assessing sarcopenia, skeletal muscle index (SMI) is considered to represent the level of muscle mass (14). Therefore, in our study, we used SMI as a standard for assessing sarcopenia. The SMI was calculated as follows: skeletal muscle area (cm^2^) /height (m^2^) (15). Due to the lack of a precise definition of sarcopenia in the Chinese population, in this study, we defined the gender-specific median SMI of patients as the cutoff value for sarcopenia. Multiple studies have demonstrated a negative correlation between skeletal muscle density (SMD) and myosteatosis. SMD was measured by obtaining the average HU of the muscles at the L3 level (16). We defined the gender-specific median SMD of patients as the cutoff value for myosteatosis.

### Statistical analysis

The clinicopathological characteristics and treatment-related adverse events (TRAEs) between the two groups were compared using the chi-squared test or Fisher’s exact test. Tumor response data were analyzed with ordinal logistic regression. Progression-free survival (PFS) and OS were estimated using the Kaplan–Meier method, with comparisons made via Log-rank tests. Factors with a *p*-value < 0.05 in univariate analysis were considered for inclusion in a multivariable Cox proportional hazards model. All *p*-values were two-sided, with values < 0.05 considered statistically significant. Statistical analyses were performed using SPSS (version 26.0), R (version 4.1.2), and GraphPad Prism (version 10.0).

## Results

### Patient characteristics

Between January 2021 and October 2024, 158 HCC patients were enrolled in this study. The baseline characteristics of included patients are presented in Table 1. The median SMI as the cutoff value for sarcopenia were 47.1 cm^2^/m^2^ in male patients and 38.2 cm^2^/m^2^ in female patients, respectively. The median SMD as the cutoff value for myosteatosis were 40.8 HU in male patients and 38.9 HU in female patients, respectively.

**Table 1:** Baseline characteristics of 158 enrolled patients.

		Sarcopenia			Myosteatosis		
Characteristics	All (*n* = 158)	Yes (*n* = 79)	No (*n* = 79)	*p* value	Yes (*n* = 80)	No (*n* = 78)	*p* value
Age, years				0.117			**0.001**
Median (range)	54 (26–79)	57 (26–79)	52 (29–78)		58 (26–79)	49 (29–76)	
< 60	111 (70.3%)	51 (64.6%)	60 (75.9%)		47 (58.8%)	64 (82.1%)	
≥ 60	47 (29.7%)	28 (35.4%)	19 (24.1%)		33 (41.2%)	14 (17.9%)	
Sex				0.822			0.873
Male	135 (85.4%)	68 (86.1%)	67 (84.8%)		68 (85.0%)	67 (85.9%)	
Female	23 (14.6%)	11 (13.9%)	12 (15.2%)		12 (15.0%)	11 (14.1%)	
BMI, kg/m^2^				**< 0.001**			0.632
Median (range)	22.8 (13.5–33.5)	20.9 (13.5–28.9)	24.0 (18.3–33.5)		22.8 (13.5–33.5)	22.7 (17.9–32.6)	
< 18.5	6 (3.8%)	5 (6.3%)	1 (1.3%)		2 (2.5%)	4 (5.1%)	
18.5–23.9	97 (61.4%)	59 (74.7%)	38 (48.1%)		51 (63.7%)	46 (59.0%)	
≥ 24	55 (34.8%)	15 (19.0%)	40 (50.6%)		27 (33.8%)	28 (35.9%)	
Karnofsky performance score				–			–
< 80	0	0	0		0	0	
≥ 80	158 (100%)	79 (100%)	79 (100%)		80 (100%)	78 (100%)	
NRS-2002 score				0.482			0.767
< 3	137 (86.7%)	67 (84.8%)	70 (88.6%)		70 (87.5%)	67 (85.9%)	
≥ 3	21 (13.3%)	12 (15.2%)	9 (11.4%)		10 (12.5%)	11 (14.1%)	
HBV				0.482			**0.003**
Positive	137 (86.7%)	70 (88.6%)	67 (84.8%)		63 (78.8%)	74 (94.9%)	
Negative	21 (13.3%)	9 (11.4%)	12 (15.2%)		17 (21.2%)	4 (5.1%)	
HCV				0.999			0.999
Positive	1 (0.6%)	1 (1.3%)	0		1 (1.3%)	0	
Negative	157 (99.4%)	78 (98.7%)	79 (100%)		79 (98.7%)	78 (100%)	
Hypertension				0.391			**0.038**
Yes	26 (16.5%)	15 (19.0%)	11 (13.9%)		18 (22.5%)	8 (10.3%)	
No	132 (83.5%)	64 (81.0%)	68 (86.1%)		62 (77.5%)	70 (89.7%)	
Diabetes				0.416			0.192
Yes	15 (9.5%)	9 (11.4%)	6 (7.6%)		10 (12.5%)	5 (6.4%)	
No	143 (90.5%)	70 (88.6%)	73 (92.4%)		70 (87.5%)	73 (93.6%)	
Cirrhosis				0.867			0.105
Yes	103 (65.2%)	51 (64.6%)	52 (65.8%)		57 (71.3%)	46 (59.0%)	
No	55 (34.8%)	28 (35.4%)	27 (34.2%)		23 (28.7%)	32 (41.0%)	
Smoking				0.699			0.490
Yes	34 (21.5%)	18 (22.8%)	16 (20.3%)		19 (23.8%)	15 (19.2%)	
No	124 (78.5%)	61 (77.2%)	63 (79.7%)		61 (76.2%)	63 (80.8%)	
Alcohol				0.685			0.051
Yes	30 (19.0%)	16 (20.3%)	14 (17.7%)		20 (25.0%)	10 (12.8%)	
No	128 (81.0%)	63 (79.7%)	65 (82.3%)		60 (75.0%)	68 (87.2%)	
Child-Pugh class				0.617			0.954
A	140 (88.6%)	71 (89.9%)	69 (87.3%)		71 (88.8%)	69 (88.5%)	
B	18 (11.4%)	8 (10.1%)	10 (12.7%)		9 (11.2%)	9 (11.5%)	
ALBI grade				**0.006**			**0.049**
0	93 (58.9%)	38 (48.1%)	55 (69.6%)		41 (51.2%)	52 (66.7%)	
1/2	65 (41.1%)	41 (51.9%)	24 (30.4%)		39 (48.8%)	26 (33.3%)	
AFP, ng/mL				0.873			0.335
≤ 400	83 (52.5%)	42 (53.2%)	41 (51.9%)		39 (48.8%)	44 (56.4%)	
> 400	75 (47.5%)	37 (46.8%)	38 (48.1%)		41 (51.2%)	34 (43.6%)	
Tumor number				0.576			0.242
Single	14 (8.9%)	6 (7.6%)	8 (10.1%)		5 (6.3%)	9 (11.5%)	
Multiple	144 (91.1%)	73 (92.4%)	71 (89.9%)		75 (93.7%)	69 (88.5%)	
Maximum tumor diameter, cm				–			–
≤ 3	0	0	0		0	0	
> 3	158 (100%)	79 (100%)	79 (100%)		80 (100%)	78 (100%)	
PVTT				0.869			0.484
Presence	59 (37.3%)	30 (38.0%)	29 (36.7%)		32 (40.0%)	27 (34.6%)	
Absence	99 (62.7%)	49 (62.0%)	50 (63.3%)		48 (60.0%)	51 (65.4%)	
Extrahepatic metastases				0.375			0.946
Presence	24 (15.2%)	10 (12.7%)	14 (17.7%)		12 (15.0%)	12 (15.4%)	
Absence	134 (84.8%)	69 (87.3%)	65 (82.3%)		68 (85.0%)	66 (84.6%)	
BCLC stage				0.874			0.522
B	81 (51.3%)	40 (50.6%)	41 (51.9%)		39 (48.8%)	42 (53.8%)	
C	77 (48.7%)	39 (49.4%)	38 (48.1%)		41 (51.2%)	36 (46.2%)	
Treatment session							
Median HAIC treatment cycles (range)	7 (2–15)	6 (2–12)	8 (3–15)	0.897	6 (2–13)	8 (3–15)	0.932
Median TKIs treatment duration, month	10.3 (0.6–19.2)	9.4 (0.6–18.9)	13.7 (1.4–19.2)	0.541	9.2 (0.6–17.7)	13.2 (2.0–19.2)	0.592
Median immunotherapy duration, month	10.4 (0.6–19.9)	9.5 (0.6–18.9)	13.9 (2.3–19.9)	0.536	9.3 (0.6–19.9)	14.0 (2.1–19.7)	0.505
Body composition variables							
SMI, cm^2^/m^2^				–			–
Male, median (range)	47.1 (26.8–73.5)	42.9 (26.8–47.1)	53.0 (47.4–73.5)		45.6 (26.8–65.9)	50.1 (32.2–73.5)	
Female, median (range)	38.2 (24.5–48.8)	35.2 (24.5–37.6)	42.6 (38.2–48.8)		36.0 (24.5–48.8)	40.9 (35.2–48.0)	
SMD, HU				–			–
Male, median (range)	40.8 (23.3–57.6)	39.5 (23.3–57.6)	42.7 (30.7–56.8)		36.5 (23.3–40.8)	45.7 (40.9–57.6)	
Female, median (range)	38.9 (27.6–49.4)	36.1 (27.6–49.4)	42.9 (30.9–49.0)		35.4 (27.6–38.9)	45.3 (39.0–49.4)	

BMI, body mass index; NRS, nutritional risk screening; HBV, hepatitis B virus; HCV hepatitis C virus; ALBI, albumin-bilirubin; AFP, alpha-fetoprotein; PVTT, portal vein tumor thrombus; BCLC stage, Barcelona Clinic Liver Cancer stage; HAIC, hepatic artery infusion chemotherapy; TKIs, tyrosine kinase inhibitors; SMI, skeletal muscle index; SMD, skeletal muscle density. Bold values indicate *p*-values less than 0.05.

There were no significant differences between the sarcopenia group and non-sarcopenia group about most epidemiologic factors such as age of diagnosis (*p* = 0.117), gender distribution (*p* = 0.822), nutritional risk screening 2002 (NRS-2002) scores (*p* = 0.482), hepatitis B virus infection (*p* = 0.482), hepatitis C virus infection (*p* = 0.999), hypertension (*p* = 0.391), diabetes (*p* = 0.416), smoking history (*p* = 0.699), and alcohol history (*p* = 0.685). Similarly, the majority of liver function characteristics such as cirrhosis (*p* = 0.867) and Child-Pugh liver function grading (*p* = 0.617) were insignificantly different. Moreover, several characteristics of liver tumors such as AFP levels (*p* = 0.873), tumor numbers (*p* = 0.576), portal vein tumor thrombus (*p* = 0.869), extrahepatic metastases (*p* = 0.375), and BCLC stage (*p* = 0.874) were similar between two groups. The two groups exhibited comparable median HAIC treatment cycles (*p* = 0.897), TKI treatment duration (*p* = 0.541), and immunotherapy duration (*p* = 0.536), with no statistically significant differences. However, patients in the sarcopenia group had lower BMI (*p* < 0.001) and higher ALBI ratio (*p* = 0.006) compared those in the non- sarcopenia group.

In addition, compared to the patients in the non-myosteatosis group, patients of myosteatosis group had older ages (*p* = 0.001), lower hepatitis B virus infection (*p* = 0.003), more hypertension history (*p* = 0.038), and higher ALBI ratio (*p* = 0.049).

### Therapeutic efficacy

The tumor responses of patients in different groups are presented in Table 2. According to the mRECIST criteria, the overall response rate (ORR) was 53.4% and the disease control rate (DCR) was 77.9% in all patients, respectively. No statistical differences in ORR or DCR were observed between the sarcopenia group and the non-sarcopenia group or between the myosteatosis group and the non-myosteatosis group.

**Table 2:** Summary of response.

		Sarcopenia			Myosteatosis		
mRECIST	All (*n* = 158)	Yes (*n* = 79)	No (*n* = 79)	*p* value	Yes (*n* = 80)	No (*n* = 78)	*p* value
CR	3 (1.9%)	1 (1.3%)	2 (2.5%)	0.561	2 (2.5%)	1 (1.3%)	0.576
PR	81 (51.3%)	41 (51.9%)	40 (50.6%)	0.874	41 (51.2%)	40 (51.3%)	0.997
SD	39 (24.7%)	18 (22.8%)	21 (26.6%)	0.580	19 (23.8%)	20 (25.6%)	0.783
PD	35 (22.1%)	19 (24.1%)	16 (20.3%)	0.565	18 (22.5%)	17 (21.8%)	0.915
ORR	84 (53.2%)	42 (53.2%)	42 (53.2%)	0.999	43 (53.7%)	41 (52.6%)	0.881
DCR	123 (77.9%)	60 (75.9%)	63 (79.7%)	0.565	62 (77.5%)	61 (78.2%)	0.915

mRECIST, modified Response Evaluation Criteria in Solid Tumors; CR, complete response; PR, partial response; SD, stable disease; PD, progressive disease; ORR, overall response rate; DCR, disease control rate.

### Safety

There was no treatment-related death in enrolled patients and TRAEs are listed in Table 3. TRAEs were observed in 98.7% of patients in the sarcopenia group and 96.2% of patients in the non-sarcopenia group. In the sarcopenia group, the most frequently reported TRAEs were fatigue (59.5%), hypoalbuminemia (55.7%), and elevated ALT levels (45.6%). Similarly, the most common TRAEs in the non-sarcopenia group were hypoalbuminemia (53.2%), fatigue (50.6%), and nausea (48.1%). Any grade of nausea (30.4% vs. 48.1%, *p* = 0.023) and grade 3/4 diarrhea (1.3% vs. 10.1%, *p* = 0.034) occurred less frequently in the sarcopenia group than in the non-sarcopenia group.

**Table 3:** Treatment-related adverse events in enrolled patients.

	Sarcopenia			Myosteatosis		
	Yes (*n* = 79)	No (*n* = 79)	*p* value	Yes (*n* = 80)	No (*n* = 78)	*p* value
TRAE						
Any grade	78 (98.7%)	77 (96.2%)	0.313	79 (98.8%)	75 (96.2%)	0.364
Grade 3/4	40 (50.6%)	46 (58.2%)	0.338	44 (55.0%)	42 (53.8%)	0.884
Neutropenia						
Any grade	26 (32.9%)	26 (32.9%)	1.000	29 (36.3%)	23 (29.5%)	0.366
Grade 3/4	5 (6.3%)	3 (3.8%)	0.469	5 (6.3%)	3 (3.8%)	0.720
Thrombocytopenia						
Any grade	28 (35.4%)	21 (26.6%)	0.229	23 (28.7%)	26 (33.3%)	0.533
Grade 3/4	5 (6.3%)	2 (2.5%)	0.248	4 (5.0%)	3 (3.8%)	0.999
Fatigue						
Any grade	47 (59.5%)	40 (50.6%)	0.263	43 (53.8%)	44 (56.4%)	0.737
Grade 3/4	0	0	–	0	0	–
Hypertension						
Any grade	30 (38.0%)	24 (30.4%)	0.314	25 (31.3%)	29 (37.2%)	0.432
Grade 3/4	3 (3.8%)	1 (1.3%)	0.620	2 (2.5%)	2 (2.6%)	0.999
Weight loss						
Any grade	24 (30.4%)	25 (31.6%)	0.863	28 (35.0%)	21 (26.9%)	0.272
Grade 3/4	2 (2.5%)	3 (3.8%)	0.999	3 (3.8%)	2 (2.6%)	0.999
Hand-foot skin reaction						
Any grade	22 (27.8%)	31 (39.2%)	0.129	28 (35.0%)	25 (32.1%)	0.695
Grade 3/4	5 (6.3%)	6 (7.6%)	0.755	6 (7.5%)	5 (6.4%)	0.788
Rash						
Any grade	9 (11.4%)	8 (10.1%)	0.797	8 (10.0%)	9 (11.5%)	0.755
Grade 3/4	0	3 (3.8%)	0.245	2 (2.5%)	1 (1.3%)	0.999
Nausea						
Any grade	24 (30.4%)	38 (48.1%)	**0.023**	28 (35.0%)	34 (43.6%)	0.269
Grade 3/4	5 (6.3%)	3 (3.8%)	0.719	4 (5.0%)	4 (5.1%)	0.999
Vomiting						
Any grade	18 (22.8%)	22 (27.8%)	0.464	19 (23.8%)	21 (26.0%)	0.647
Grade 3/4	1 (1.3%)	1 (1.3%)	1.000	1 (1.3%)	1 (1.3%)	0.999
Diarrhea						
Any grade	13 (16.5%)	22 (27.8%)	0.085	14 (17.5%)	21 (26.9%)	0.154
Grade 3/4	1 (1.3%)	8 (10.1%)	**0.034**	3 (3.8%)	6 (7.7%)	0.325
Abdominal pain						
Any grade	25 (31.6%)	27 (34.2%)	0.735	25 (31.3%)	27 (34.6%)	0.653
Grade 3/4	4 (5.1%)	6 (7.6%)	0.513	3 (3.8%)	7 (9.0%)	0.307
Sensory neuropathy						
Any grade	13 (16.5%)	13 (16.5%)	1.000	11 (13.8%)	15 (19.2%)	0.353
Grade 3/4	1 (1.3%)	1 (1.3%)	1.000	1 (1.3%)	1 (1.3%)	0.999
Proteinuria						
Any grade	20 (25.3%)	14 (17.7%)	0.245	20 (25.0%)	14 (17.9%)	0.281
Grade 3/4	1 (1.3%)	1 (1.3%)	1.000	2 (2.5%)	0	0.497
Elevated ALT						
Any grade	36 (45.6%)	35 (44.3%)	0.873	37 (46.3%)	34 (43.6%)	0.737
Grade 3/4	10 (12.7%)	9 (11.4%)	0.807	9 (11.3%)	10 (12.8%)	0.762
Elevated AST						
Any grade	35 (44.3%)	31 (39.2%)	0.519	35 (43.8%)	31 (39.7%)	0.610
Grade 3/4	15 (19.0%)	17 (21.5%)	0.692	14 (17.5%)	18 (23.1%)	0.383
Hyperbilirubinemia						
Any grade	30 (38.0%)	28 (35.4%)	0.741	30 (37.5%)	28 (35.9%)	0.834
Grade 3/4	4 (5.1%)	2 (2.5%)	0.681	2 (2.5%)	4 (5.1%)	0.440
Hypoalbuminemia						
Any grade	44 (55.7%)	42 (53.2%)	0.749	45 (56.3%)	41 (52.6%)	0.642
Grade 3/4	1 (1.3%)	2 (2.5%)	0.999	1 (1.3%)	2 (2.6%)	0.618
Fever						
Any grade	11 (13.9%)	10 (12.7%)	0.815	13 (16.3%)	8 (10.3%)	0.267
Grade 3/4	0	0	–	0	0	–
Anemia						
Any grade	33 (41.8%)	25 (31.6%)	0.187	29 (36.3%)	29 (37.2%)	0.904
Grade 3/4	3 (3.8%)	0	0.245	2 (2.5%)	1 (1.3%)	0.999
Elevated creatinine						
Any grade	4 (5.1%)	6 (7.6%)	0.513	7 (8.8%)	3 (3.8%)	0.348
Grade 3/4	0	2 (2.5%)	0.497	2 (2.5%)	0	0.497

TRAE, treatment-related adverse events; ALT, alanine aminotransferase; AST, aspartate aminotransferase. Bold values indicate *p*-values less than 0.05.

TRAEs were reported in 98.8% of patients in the myosteatosis group and 96.2% of patients in the non-myosteatosis group. In the myosteatosis group, the most common TRAEs included hypoalbuminemia (56.3%), fatigue (53.8%), and elevated ALT levels (46.3%). Similarly, in the non-myosteatosis group, the most frequent TRAEs were hypoalbuminemia (52.6%), fatigue (56.4%), nausea (43.6%), and elevated ALT levels (43.6%). No significant difference in TRAEs incidence was observed between the two groups.

### Survival outcomes

**Figure 2:**
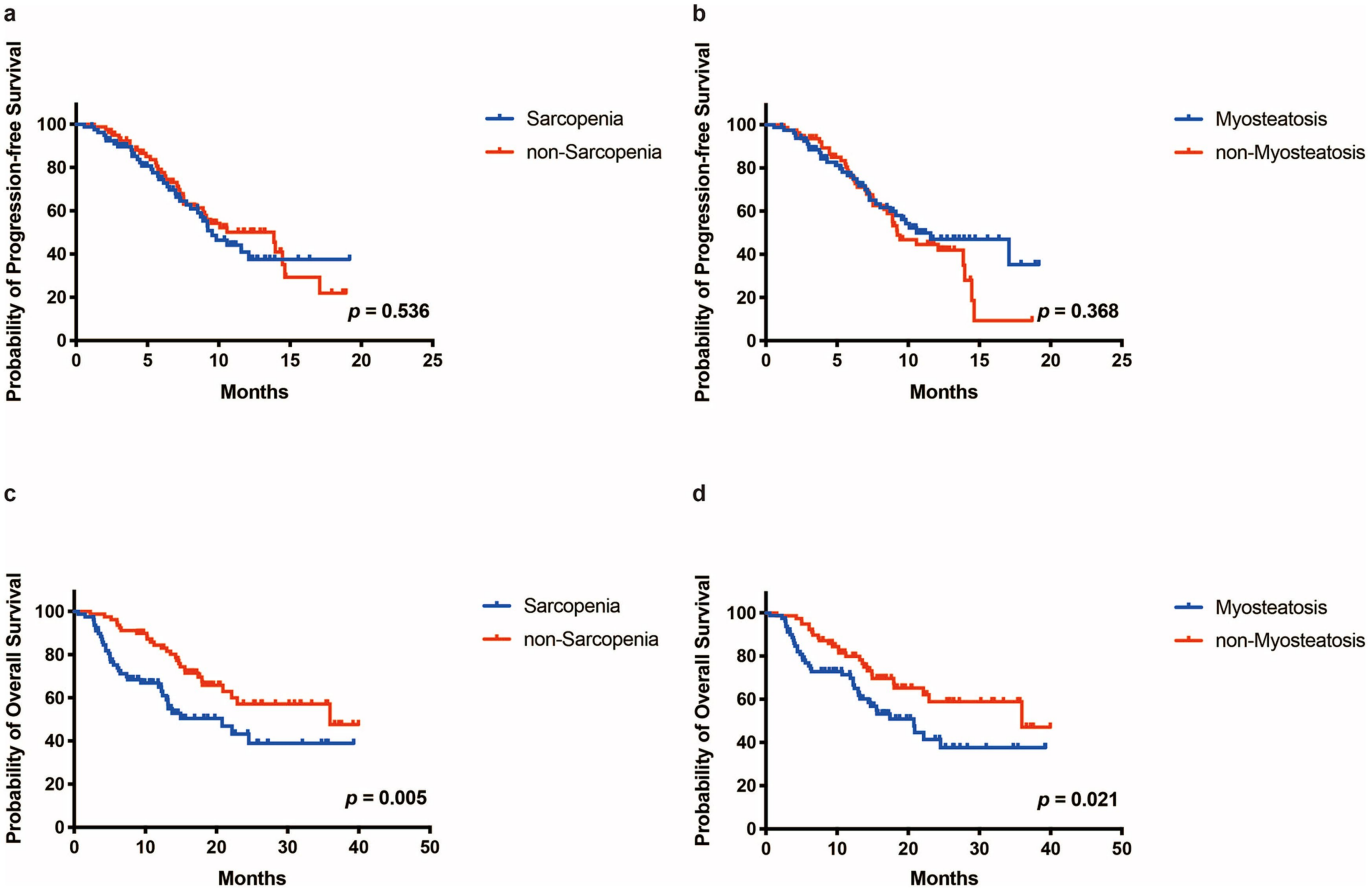
The Progressione-free survival (PFS) and overall survival (OS) of patients in different groups. **(a,b)** PFS curve of patients in the sarcopenia and the non-sarcopenia group. **(c,d)** OS curve of patients in the sarcopenia and the non-sarcopenia group.

The median follow-up time was 16.99 months (2.83–41.30 months). No significant differences in median PFS were observed between the sarcopenia and the non-sarcopenia groups (9.53 vs. 13.87 months, *p* = 0.536, Figure 2a) or between the myosteatosis and the non-myosteatosis groups (10.57 vs. 9.23 months, *p* = 0.368, Figure 2b). Among patients in the sarcopenia group, the PFS rates at 3-, 6-, and 12-month were 89.6, 74.4, and 37.5%, respectively. In comparison, the corresponding PFS rates for patients in the non-sarcopenia group were 93.6, 77.7, and 50.1%. In the myosteatosis group, the PFS rates at 3-, 6-, and 12-month were 89.8, 76.5, and 47.0%, respectively. Conversely, the corresponding PFS rates in the non-myosteatosis group were 93.5, 75.8, and 44.6%.

The median OS in the sarcopenia group was 20.80 months compared to 35.97 months in the non-sarcopenia group (*p* = 0.005, Figure 2c). Among patients in the sarcopenia group, the OS rates at 3-, 6-, and 12-month were 92.4, 75.2, and 65.0%, respectively. In contrast, the corresponding OS rates for patients in the non-sarcopenia group were 98.7, 94.9, and 84.4%. Similarly, the median OS of the myosteatosis group (20.80 months) was significantly shorter than that of the non-myosteatosis group (35.97 months) (*p* = 0.021, Figure 2d). In the myosteatosis group, the OS rates at 3-, 6-, and 12-month were 92.5, 75.5, and 69.8%, respectively. By comparison, the corresponding OS rates in the non-myosteatosis group were 98.7, 94.9, and 79.9%.

### Univariate and multivariate analysis of survival

The results of the univariate and multivariate analyses of PFS and OS are presented in Supplementary Table 1 and Table 4, respectively. Multivariate analysis demonstrated that the independent risk factor for PFS was AFP level (≤ 400 ng/mL vs. > 400 ng/mL, HR: 0.592, 95% CI: 0.361–0.971; *p* = 0.038). Multivariate analysis for OS showed that gender distribution (male vs. female, HR: 0.513, 95% CI: 0.270–0.975; *p* = 0.042), BMI (< 18.5 vs. 18.5–23.9 vs. ≥ 24, HR: 1.896, 95% CI: 1.025–3.506; *p* = 0.041), AFP level (≤ 400 ng/mL vs. > 400 ng/mL, HR: 0.484, 95% CI: 0.291–0.805; *p* = 0.005), tumor number (single vs. multiple, HR: 0.215, 95% CI: 0.051–0.917; *p* = 0.038), and sarcopenia (yes vs. no, HR: 0.527, 95% CI: 0.311–0.893; *p* = 0.017) were prognostic factors. Although myosteatosis was a significant factor affecting OS in univariate analysis (*p* = 0.021), there was no statistical difference in multivariate analysis (*p* = 0.109).

**Table 4:** Univariate and multivariate analyses of overall survival.

Variables	Median OS (months)	Univariate analysis	Multivariate analysis		
		*p* value	*p* value	HR	95% CI
Age, years		0.328			
< 60	Not reached				
≥ 60	22.13				
Sex		**0.044**	**0.042**	0.513	0.270–0.975
Male	35.97				
Female	13.00				
BMI, kg/m^2^		**0.018**	**0.041**	1.896	1.025–3.506
< 18.5	11.87				
18.5–23.9	19.97				
≥ 24	Not reached				
NRS-2002 score		0.188			
< 3	24.53				
≥ 3	13.17				
HBV		0.254			
Positive	22.17				
Negative	Not reached				
Hypertension		0.862			
Yes	22.17				
No	35.97				
Diabetes		0.281			
Yes	Not reached				
No	22.93				
Cirrhosis		0.593			
Yes	22.17				
No	24.53				
Smoking		0.557			
Yes	Not reached				
No	22.17				
Alcohol		0.736			
Yes	Not reached				
No	24.53				
Child-Pugh class		0.142			
A	24.53				
B	17.43				
ALBI grade		0.063			
0	35.97				
1/2	20.80				
AFP, ng/mL		**0.004**	**0.005**	0.484	0.291–0.805
≤ 400	Not reached				
> 400	15.53				
Tumor number		**0.024**	**0.038**	0.215	0.051–0.917
Single	Not reached				
Multiple	22.13				
PVTT		0.447			
Presence	35.97				
Absence	22.17				
Extrahepatic metastases		0.068			
Presence	17.97				
Absence	35.97				
BCLC stage		0.065			
B	Not reached				
C	20.90				
Sarcopenia		**0.005**	**0.017**	0.527	0.311–0.893
Yes	20.80				
No	35.97				
Myosteatosis		**0.021**	0.109	0.660	0.397–1.097
Yes	20.80				
No	35.97				

OS, overall survival; BMI, body mass index; NRS, nutritional risk screening; HBV, hepatitis B virus; HCV hepatitis C virus; ALBI, albumin-bilirubin; AFP, alpha-fetoprotein; PVTT, portal vein tumor thrombus; BCLC stage, Barcelona Clinic Liver Cancer stage. Bold values indicate *p*-values less than 0.05.

## Discussion

Recent studies by our team and other researchers have indicated that HAIC combined with targeted therapy and immunotherapy can significantly improve the prognosis of patients with advanced HCC (3, 4). But the early identification of patients who may benefit from this triple treatment remains unclear. Previous studies have reported associations between changes in body composition and HCC prognosis (12, 15, 17, 18). However, these studies have several limitations: (1) most focused on the relationship between body composition and a single treatment modality, such as surgery, interventional therapy, targeted therapy, immunotherapy, or radiotherapy; (2) the majority used sarcopenia as the primary indicator, which primarily reflects changes in muscle quantity and does not accurately capture alterations in muscle quality; and (3) although one study investigated the relationship between sarcopenia and the prognosis of HCC patients treated with interventional therapy combined with targeted therapy and immunotherapy, it combined two distinct interventional approaches, transarterial chemoembolization (TACE) and HAIC, and focused solely on sarcopenia (19).

According to European and Asian guidelines, sarcopenia is commonly regarded as a key indicator for evaluating malnutrition. Sarcopenia can be categorized into primary and secondary types, with secondary sarcopenia commonly resulting from conditions such as cirrhosis or tumors (10, 20). However, the exact definition of sarcopenia remains unclear. Some studies differentiate sarcopenia based on a specific value of SMI relative to the patient’s BMI (21). More commonly, studies use the median SMI of the study population for classification (12, 15, 19, 22). In this study, to eliminate ethnic differences, sarcopenia was defined using gender-specific SMI medians.

Recent studies suggest that myosteatosis can also reflect muscle quality in patients and is significantly associated with poor prognosis in conditions such as cancer and abdominal trauma (15, 16, 22). SMD is a commonly used indicator for evaluating myosteatosis. Similar to sarcopenia, the precise definition for distinguishing myosteatosis remains unclear (15). In this study, myosteatosis was defined using gender-specific SMD medians.

Our study found that patients in the sarcopenia group had a lower BMI. Although most studies suggest that BMI is not related to sarcopenia, some studies indicate that sarcopenic patients tend to have a lower BMI (23). A possible explanation is that the lower BMI in sarcopenic patients may be due to a reduction in muscle mass leading to weight loss, while fat mass remains unchanged or increases (24). On the other hand, our study found that patients suffering from sarcopenia or myosteatosis had a higher ALBI ratio. HCC patients often experience malnutrition due to factors such as reduced appetite and impaired nutrient absorption, which can lead to sarcopenia, myosteatosis, and decreased serum albumin levels. An elevated ALBI ratio in this context may reflect poor nutritional status and muscle loss (25). Additionally, patients with impaired liver function often experience chronic inflammation and metabolic disturbances, which not only affect the metabolism of albumin and bilirubin but may also promote muscle breakdown, further exacerbating the development of sarcopenia or myosteatosis (26). Our study also found that patients in the myosteatosis group were older. Previous research has suggested a relationship between myosteatosis and age. As individuals age, the proportion of fat in muscle tissue tends to increase. Additionally, aging is closely associated with chronic inflammation, changes in hormone levels (such as decreased growth hormone and testosterone), and metabolic dysregulation, all of which may contribute to the accumulation of fat within the muscles (23).

The results of this study show that although the incidence of disease progression (PD) was higher in the sarcopenia group compared to the non-sarcopenia group (a similar trend was observed when comparing the myosteatosis and non-myosteatosis groups), the difference was not statistically significant. The ORR and DCR were similar between the two groups. Interestingly, while some reports suggest that the ORR and DCR are lower in sarcopenia patients than in non-sarcopenia patients (20), other studies argue that the development of sarcopenia in the short term after treatment may better indicate poor treatment outcomes (12, 22). Therefore, dynamically monitoring changes in patients’ body composition, such as SMI and SMD, may be more helpful in predicting treatment efficacy.

The results of this study indicate that the types of TRAEs were similar between the sarcopenia group and the non-sarcopenia group. However, the incidence of nausea and severe diarrhea was lower in the sarcopenia group. This may be attributed to the relatively lower body fat percentage in sarcopenia patients, which can influence the distribution and metabolism of certain antitumor drugs, particularly lipophilic drugs (27). Such changes may reduce the direct effects of these drugs on the gastrointestinal tract, thereby lowering the risk of nausea. Additionally, sarcopenia patients generally have lower body weight, which may lead to adjustments in drug dosages based on body weight or body surface area. These dosage modifications could help reduce gastrointestinal side effects, including nausea and severe diarrhea (28).

Interestingly, this study found no significant difference in PFS between sarcopenia and non-sarcopenia patients following triplet therapy. Although most studies have reported an association between sarcopenia and shorter PFS, these studies typically focus on a single treatment modality, such as surgery, targeted therapy, or radiotherapy (10). The impact of sarcopenia on the efficacy of combined targeted therapy and immunotherapy remains controversial, with some literature suggesting that sarcopenia does not affect PFS in patients treated with atezolizumab and bevacizumab (20). In this study, patients received HAIC in combination with targeted therapy and immunotherapy, and potential interactions between these therapies may influence outcomes. Therefore, sarcopenia may not serve as a reliable predictor of PFS in patients undergoing triplet therapy.

Most importantly, the results of this study indicate that OS was shorter in sarcopenia patients compared to non-sarcopenia patients, with similar findings observed between myosteatosis and non-myosteatosis patients. Multivariate analysis further identified sarcopenia as a risk factor for OS. Sarcopenia and myosteatosis reflect declines in skeletal muscle quantity, quality, and strength. In HCC patients, muscle changes result from the complex interplay of factors such as nutritional deficiencies, physical inactivity, liver dysfunction, hormonal/cytokine imbalances, and immune dysregulation, all of which contribute to muscle deterioration and functional impairment. This deterioration in body composition may, in turn, negatively impact the efficacy of therapeutic interventions (29, 30). Although previous studies have suggested an association between sarcopenia and poor prognosis in HCC treatment, this study is the first to explicitly establish a correlation between body composition changes, such as sarcopenia and myosteatosis, and the prognosis of triple therapy combining HAIC, targeted therapy, and immunotherapy.

Our study has several limitations. First, as a single-center retrospective study, it requires the inclusion of a larger patient cohort to validate the conclusions. Second, the study population consisted of East Asian individuals, and further research is needed to determine whether similar conclusions can be drawn in other ethnic groups. Third, the number of female patients included in the study was relatively small, making it difficult to conduct further stratified analyses. Fourth, the targeted therapy and immunotherapy regimens in this study involved multiple drugs, and future investigations are needed to differentiate the effects of specific drugs and their associations with body composition.

## Conclusion

Our study found that sarcopenia and myosteatosis can predict poor prognosis in HCC patients undergoing HAIC combined with targeted therapy and immunotherapy, with sarcopenia identified as an independent risk factor for OS. This suggests that changes in body composition are associated with the efficacy of triple therapy in HCC. However, neither sarcopenia nor myosteatosis showed a significant association with ORR, DCR, or PFS.

## Data Availability

The raw data supporting the conclusions of this article will be made available by the authors, without undue reservation.
