# Supplementary material for: Prognostic impact of body composition in hepatocellular carcinoma patients undergoing interventional and systemic therapy
Source: Front Nutr. 2025 Apr 16;12:1586202. doi: 10.3389/fnut.2025.1586202 (PMC12040644; doi:10.3389/fnut.2025.1586202)
Supplement: Supplementary file 1 [file Table_1.docx]

**Supplement Table 1** Univariate and multivariate analyses of progression-free survival

| Variables | Median PFS (months) | Univariate analysis | Multivariate analysis | | |
| --- | --- | --- | --- | --- | --- |
|  |  | *p* value | *p* value | HR | 95% CI |
| Age, years |  | **0.016** | 0.244 | 1.460 | 0.772-2.762 |
| < 60 | 9.13 |  |  |  |  |
| ≥ 60 | Not reached |  |  |  |  |
| Sex |  | 0.999 |  |  |  |
| Male | 10.57 |  |  |  |  |
| Female | 10.07 |  |  |  |  |
| BMI, kg/m^2^ |  | 0.166 |  |  |  |
| < 18.5 | 7.00 |  |  |  |  |
| 18.5-23.9 | 8.93 |  |  |  |  |
| ≥ 24 | 13.87 |  |  |  |  |
| NRS-2002 score |  | 0.195 |  |  |  |
| < 3 | 10.57 |  |  |  |  |
| ≥ 3 | 7.00 |  |  |  |  |
| HBV |  | 0.113 |  |  |  |
| Positive | 9.43 |  |  |  |  |
| Negative | Not reached |  |  |  |  |
| Hypertension |  | 0.085 |  |  |  |
| Yes | 13.87 |  |  |  |  |
| No | 9.43 |  |  |  |  |
| Diabetes |  | 0.688 |  |  |  |
| Yes | 9.83 |  |  |  |  |
| No | 10.57 |  |  |  |  |
| Cirrhosis |  | 0.640 |  |  |  |
| Yes | 9.23 |  |  |  |  |
| No | 12.10 |  |  |  |  |
| Smoking |  | 0.274 |  |  |  |
| Yes | Not reached |  |  |  |  |
| No | 9.53 |  |  |  |  |
| Alcohol |  | 0.380 |  |  |  |
| Yes | Not reached |  |  |  |  |
| No | 9.43 |  |  |  |  |
| Child-Pugh class |  | **0.030** | 0.185 | 0.617 | 0.302-1.259 |
| A | 11.57 |  |  |  |  |
| B | 7.13 |  |  |  |  |
| ALBI grade |  | 0.313 |  |  |  |
| 0 | 11.57 |  |  |  |  |
| 1/2 | 9.23 |  |  |  |  |
| AFP, ng/mL |  | **0.001** | **0.038** | 0.592 | 0.361-0.971 |
| ≤ 400 | 13.97 |  |  |  |  |
| > 400 | 8.90 |  |  |  |  |
| Tumor number |  | 0.296 |  |  |  |
| Single | 7.73 |  |  |  |  |
| Multiple | 10.57 |  |  |  |  |
| PVTT |  | **0.001** | 0.300 | 0.566 | 0.193-1.661 |
| Presence | 7.53 |  |  |  |  |
| Absence | 17.07 |  |  |  |  |
| Extrahepatic metastases |  | **0.001** | 0.121 | 0.470 | 0.181-1.219 |
| Presence | 6.13 |  |  |  |  |
| Absence | 13.87 |  |  |  |  |
| BCLC stage |  | **0.001** | 0.132 | 0.386 | 0.112-1.330 |
| B | Not reached |  |  |  |  |
| C | 7.33 |  |  |  |  |
| Sarcopenia |  | 0.536 |  |  |  |
| Yes | 9.53 |  |  |  |  |
| No | 13.87 |  |  |  |  |
| Myosteatosis |  | 0.368 |  |  |  |
| Yes | 10.57 |  |  |  |  |
| No | 9.23 |  |  |  |  |

Abbreviations: PFS, progression-free survival; BMI, body mass index; NRS, nutritional risk screening; HBV, hepatitis B virus; HCV hepatitis C virus; ALBI, albumin-bilirubin; AFP, alpha-fetoprotein; PVTT, portal vein tumor thrombus; BCLC stage, Barcelona Clinic Liver Cancer stage.

Bold values ​​indicate *p*-values ​​less than 0.05.
